# Advances in ultrasonography: image formation and quality assessment

**DOI:** 10.1007/s10396-021-01140-z

**Published:** 2021-10-20

**Authors:** Hideyuki Hasegawa

**Affiliations:** Faculty of Engineering, University of Toyama, 3190 Gofuku, Toyama, 930-8555 Japan

**Keywords:** Medical ultrasound image, Adaptive imaging, Image quality

## Abstract

Delay-and-sum (DAS) beamforming is widely used for generation of B-mode images from echo signals obtained with an array probe composed of transducer elements. However, the resolution and contrast achieved with DAS beamforming are determined by the physical specifications of the array, e.g., size and pitch of elements. To overcome this limitation, adaptive imaging methods have recently been explored extensively thanks to the dissemination of digital and programmable ultrasound systems. On the other hand, it is also important to evaluate the performance of such adaptive imaging methods quantitatively to validate whether the modification of the image characteristics resulting from the developed method is appropriate. Since many adaptive imaging methods have been developed and they often alter image characteristics, attempts have also been made to update the methods for quantitative assessment of image quality. This article provides a review of recent developments in adaptive imaging and image quality assessment.

## Introduction

This article provides a review of recent developments in medical ultrasound imaging. This review starts with a brief description of the beamforming process in medical ultrasound imaging. Then, rather “traditional” adaptive imaging methods that enhance some image quality evaluation metrics, i.e., resolution, contrast, and contrast-to-noise ratio (CNR), are described, and they are compared in terms of performance. Furthermore, the limitations of such traditional adaptive imaging methods are discussed, and new evaluation metrics that have been developed recently for more appropriate evaluation of image quality are described. Finally, recent studies that tackle the limitations of traditional adaptive imaging methods will be introduced, and a few examples of the results obtained with the methods developed in such studies are also shown.

## Beamforming

**Fig. 1:**
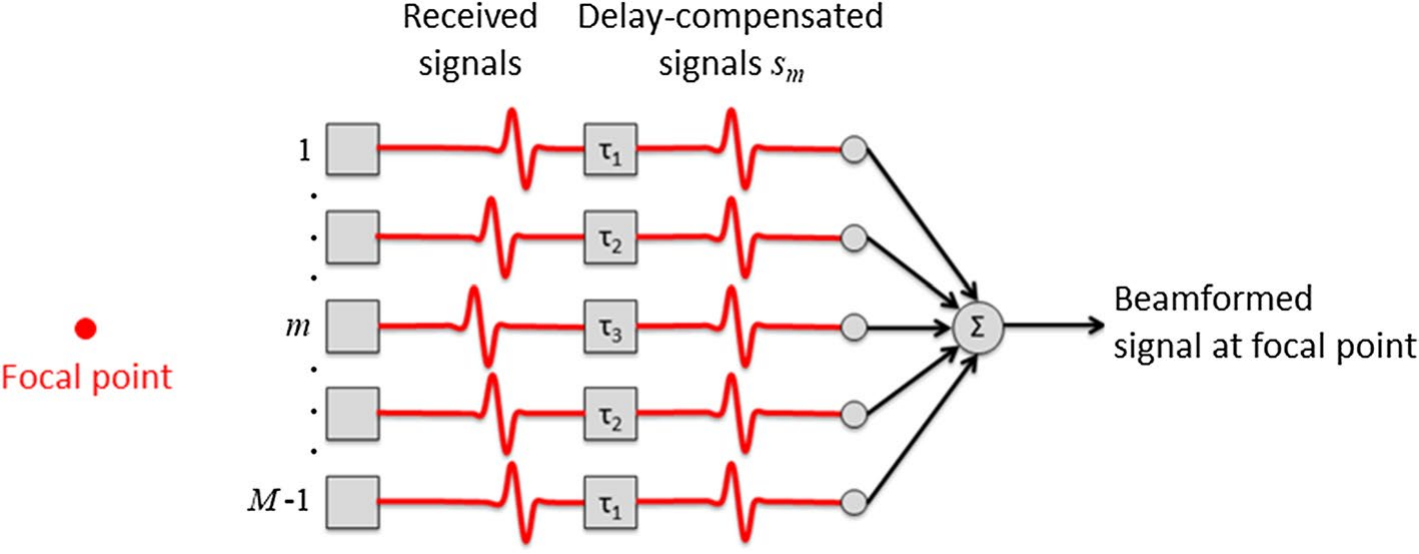
Illustration of delay-and-sum (DAS) beamforming

Beamforming is an indispensable part of the process for generating ultrasound images. Although the beamforming process is important for controlling the ultrasonic field in transmission, the beamforming process in reception is discussed in this article. Delay-and-sum (DAS) beamforming is well known and widely used in clinical ultrasound scanners. As illustrated in Fig. 1, a DAS beamformer creates a beamformed signal at a point of interest (focal point). By setting a focal point, the beamformer can estimate the propagation time between the focal point and each transducer element by assuming the speed of sound in the propagation medium. By delaying the echo signals received by individual elements (channel signals) based on the estimated propagation time, echoes from the focal point become in phase. Consequently, the echo from the focal point is enhanced, and out-of-focus echoes are suppressed, by accumulating the delayed signals across the aperture. This procedure is expressed as

1 $$p_{\rm DAS} = \frac{1}{M}\mathop \sum \limits_{m = 0}^{M - 1} s_{m} ,$$

where $p_{\rm DAS}$ is the output of the DAS beamformer, $s_{m}$ is the delay-compensated radio-frequency (RF) echo signal received by the $m$-th transducer element, and $M$ denotes the number of elements in the receiving aperture. The spatial resolution of the DAS beamformer is limited by the aperture size, and side and grating lobes cannot be suppressed perfectly due to the finite aperture size and insufficient element pitch.

A nonlinear beamformer, namely, delay multiply-and-sum (DMAS) beamformer, which was first developed for microwave radar imaging of breast cancers [1], was also introduced in medical ultrasound imaging [2, 3]. The output of the DMAS beamformer $p_{\rm {DMAS}}$ is expressed as

2 $$p_{\rm {DMAS}} = \mathop \sum \limits_{i = 1}^{M - 1} \mathop \sum \limits_{j = i + 1}^{M}{\rm sign}\left( {s_{i} \cdot s_{j} } \right) \cdot \sqrt {\left| {s_{i} \cdot s_{j} } \right|} .$$

With respect to the output of the DMAS beamformer, DC and $2f_{0}$ components, where $f_{0}$ is the center frequency of $s_{m}$, are generated by multiplication of the signals. Therefore, a band-pass filter is required to obtain the final output of the beamformer:

3 $$p_{\rm {F-DMAS}} = {\rm {BPF}}\left[ {p_{\rm {DMAS}} } \right],$$

where ${\text{BPF}}\left[ \cdot \right]$ denotes band-pass filtering. This beamformer is called a filtered-delay-and-multiply-sum (FDMAS) beamformer. The FDMAS beamformer realizes superior resolution and contrast in comparison with the DAS beamformer because the multiply-and-sum operation corresponds to evaluation of signal coherence [2]. The FDMAS method is also used for coherent compounding in plane-wave imaging [4–6].

## Adaptive imaging methods

To overcome the limitation of the DAS beamformer, various adaptive imaging methods with data-dependent output have been introduced in ultrasound imaging. Since numerous imaging methods have been developed, some representative methods are described in this article to discuss recent trends in adaptive imaging.

### Coherence-based adaptive imaging

The quality of an ultrasonic image is degraded by phase aberrations from an inhomogeneous distribution of sound speeds in a biological tissue. The coherence factor (CF) [7–9] was developed for evaluation of such degradation in image quality. It was defined as the ratio of the coherent energy to the total received energy [7]:

4 $$CF = \frac{{\left| {\frac{1}{M}\sum\nolimits_{m = 0}^{M - 1} {\tilde{s}_{m} } } \right|^{2} }}{{\frac{1}{M}\sum\nolimits_{m = 0}^{M - 1} {\left| {\tilde{s}_{m} } \right|^{2} } }}$$

where $\tilde{s}_{m}$ is the complex analytic signal of the delay-compensated RF signal received by the $m$-th elements in the receiving aperture. Li PC and Li ML suppress incoherent signals by weighting DAS beamformed signals by CF as [10]:

5 $$p_{\rm {CF}} = CF \cdot p_{\rm {DAS}} ,$$

where $p_{\rm {CF}}$ is the output of the beamformer with CF weighting. The resolution and contrast of an ultrasonic image are improved by suppressing incoherent components. The coherence among channel signals has also been evaluated using the phase of the channel signals, namely, phase coherence factor [11–13]. Similarly to CF, incoherent signals can be suppressed by weighting DAS beamformed signals by the phase coherence factor in the same way as Eq. (5).

As can be seen in Eq. (1), CF of a perfectly coherent signal will be 1, and that of a perfectly incoherent signal will be 0. To further increase the difference between coherent and incoherent signals, the mean-to-standard-deviation (MSD) factor [14] and signal-to-noise-ratio (SNR) factor [15] were introduced. The CF value for a perfectly coherent signal is limited to 1 because CF is defined as the ratio of coherent energy to “total energy”. By evaluating the ratio of coherent energy to energy of “noise” (incoherent component), MSD and SNR factors will be infinite with respect to a perfectly coherent signal. However, infinite amplification of the signal makes the beamformer output unstable. Therefore, a stabilization term is introduced in the SNR factor as

6 $$SNR = \frac{{\left| {\tilde{p}_{\rm {DAS}} } \right|^{2} }}{{\frac{1}{M}\mathop \sum \nolimits_{m = 0}^{M - 1} \left| {\tilde{p}_{m} - \tilde{p}_{\rm {DAS}} } \right|^{2} - \gamma \cdot \left| {\tilde{p}_{\rm {DAS}} } \right|^{2} }},$$

where $\gamma$ is a stabilization parameter and $\tilde{p}_{\rm {DAS}}$ is the output of the DAS beamformer obtained using complex analytic signals of channel RF signals. The numerator in Eq. (6) is the same as that in Eq. (4). The beamformer output $p_{\rm {SNR}}$ with SNR weighting is obtained in the same way as that with CF weighting shown in Eq. (5).

**Fig. 2:**
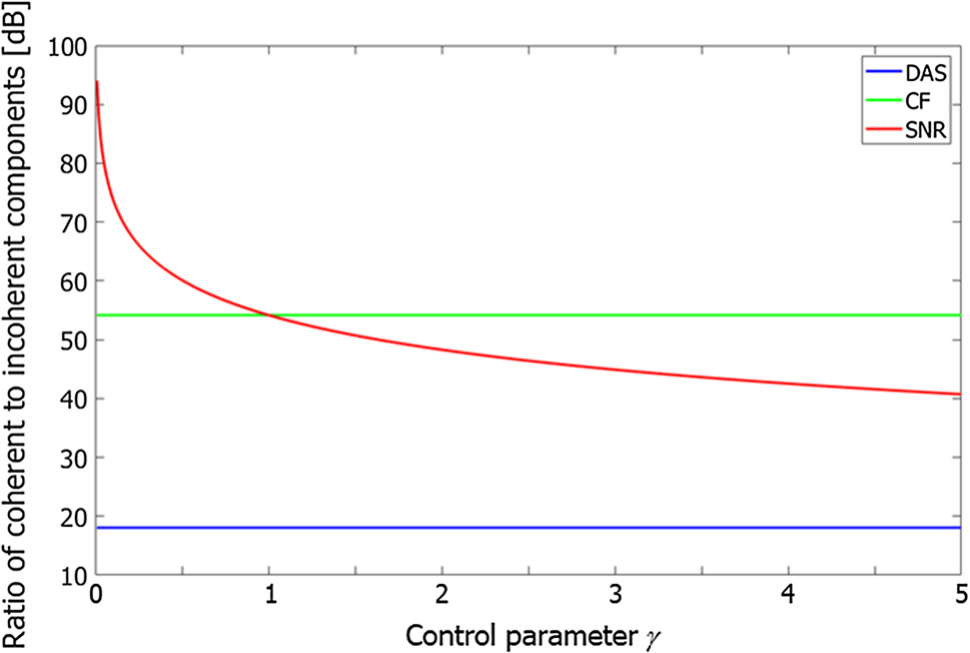
Theoretical ratios of coherent to incoherent components

By weighting DAS beamformed signals with the MSD or SNR factor, contrast between coherent and incoherent components can be increased in comparison with CF. The difference between coherent and incoherent signals, in particular, is amplified to infinite using the SNR factor when the stabilization parameter $\gamma$ is zero. However, such amplification would not be infinite because the aperture size is finite in real situations. The ratio of the power of the coherent component to that of the incoherent components in the output of each beamformer is expressed as follows [15]:

7 $$R_{\rm {DAS}} = M\left| {\frac{p}{{\sigma_{n} }}} \right|^{2} ,$$

8 $$R_{\rm {CF}} = M^{3} \left| {\frac{p}{{\sigma_{n} }}} \right|^{2} ,$$

9 $$R_{\rm {SNR}} = \frac{{M\left| {M + \left( {\gamma - 1} \right)} \right|^{2} }}{{\gamma^{2} }}\left| {\frac{p}{{\sigma_{n} }}} \right|^{2} ,$$

where $R_{\rm {DAS}}$, $R_{\rm {CF}}$, and $R_{\rm {SNR}}$ are the ratios for the DAS beamformer, DAS beamformer with CF weighting, and that with SNR weighting, respectively, $M$ is the number of elements in the receiving aperture, $p$ is the amplitude of the echo from the focal point, and ${\sigma }_{n}$ is the mean amplitude of noise. Figure 2 shows the theoretical ratios of coherent to incoherent components obtained by setting $M$ in Eqs. (7)-(9) at 64. Note that the values plotted in Fig. 2 were normalized by $\left| {p/\sigma_{n} } \right|^{2}$, and the minimum value of the stabilization parameter $\gamma$ for the SNR factor was set at 0.01. As can be seen in Fig. 2, CF and the SNR factor significantly increase the difference between coherent and incoherent components. The SNR factor is identical to CF when $\gamma = 1.$ Compared with CF, the SNR factor amplifies the ratio of coherent to incoherent components when $\gamma < 1.$

### Adaptive beamforming

The minimum variance (MV) beamformer [16–23] is a representative method for adaptive beamforming. In DAS beamforming, the apodization scheme, which applies weights to channel signals, is in general used to lower the side-lobe level. The MV beamformer adaptively determines the weights depending on the received channel signals, while the apodization weights in DAS beamforming are fixed. Also, the apodization weights in DAS beamforming are real values controlling only amplitudes of channel signals. On the other hand, the weights in MV beamforming are complex-valued, and the MV beamformer also controls time delays applied to channel signals so that the output power becomes minimum while keeping the all-path characteristics with respect to the desired direction (focal point). This problem is expressed as follows: The channel echo signal is expressed in a vector form as

10 $$\tilde{\user2{s}} = \left[ {\tilde{s}_{0} \tilde{s}_{1} \tilde{s}_{2} \ldots \tilde{s}_{M - 1} } \right]^{{\text{T}}} ,$$

where ^T^ denotes transpose. The beamformer weights ${w}_{m}$ are also expressed in a vector form as

11 $${\varvec{w}} = \left[ {w_{0} w_{1} w_{2} \ldots w_{M - 1} } \right]^{{\text{T}}} .$$

Since the output of the MV beamformer is expressed as $p_{\rm {MV}} = {\varvec{w}}^{{\text{H}}} \cdot \tilde{\user2{s}}$, the expected power of the MV beamformer is expressed as

12 $$E\left[ {\left| {p_{{{\text{MV}}}} } \right|^{2} } \right] = {\varvec{w}}^{H} E\left[ {\tilde{\user2{s}}\tilde{\user2{s}}}^{H} \right]{\varvec{w}} = {\varvec{w}}^{H} {\varvec{Rw}},$$

where *H*, $E\left[\bullet \right],$ and ***R*** denote the Hermitian transpose, expectation, and covariance matrix, respectively. The minimization problem is described as

13 $$\widehat{{\varvec{w}}}_{{{\text{MV}}}} = \arg \mathop {\min }\limits_{{\varvec{w}}} E\left[ {\left| {\user2{w}\tilde{\user2{s}}} \right|^{2} } \right] = \arg \mathop {\min }\limits_{{\varvec{w}}} {\varvec{w}}^{H} {\varvec{Rw}},{\text{ subject to}}~{\varvec{w}}^{H} {\varvec{a}} = 1,$$

where ***a*** is a steering vector. The solution to this problem is given by

14 $$\widehat{{\varvec{w}}}_{{{\text{MV}}}} = \frac{{{\varvec{R}}^{ - 1} {\mathbf{a}}}}{{{\varvec{a}}^{{\text{H}}} {\varvec{R}}^{ - 1} {\varvec{a}}}}.$$

The steering vector ***a*** becomes a vector of ones when the differences in time delays of channel signals due to the differences in propagation distances between elements and focal point are compensated. Also, diagonal loading, which adds small values to the diagonal components of a matrix, is applied to covariance matrix ***R*** to stabilize the beamformer output.

Various attempts have been made to improve the performance of the MV beamformer. The forward–backward estimation of a covariance matrix improved image contrast and robustness in MV beamforming [24]. Eigenvalue decomposition of the covariance matrix was used in MV beamforming and applied to imaging of hard tissues for better delineation of edges [25, 26], and a covariance matrix obtained from combinations of different sub-arrays improved image contrast in MV beamforming [27]. Also, the MV beamformer was combined with coherence factors to improve resolution and contrast further [28–30]. Furthermore, Wiener filtering was used in the beamforming process and coherence estimation [31–33]. The MV beamformer can also be applied to determination of the weights in coherent compounding of plane-wave images obtained at different steering angles [34–36].

## Assessment of image quality

**Fig. 3:**
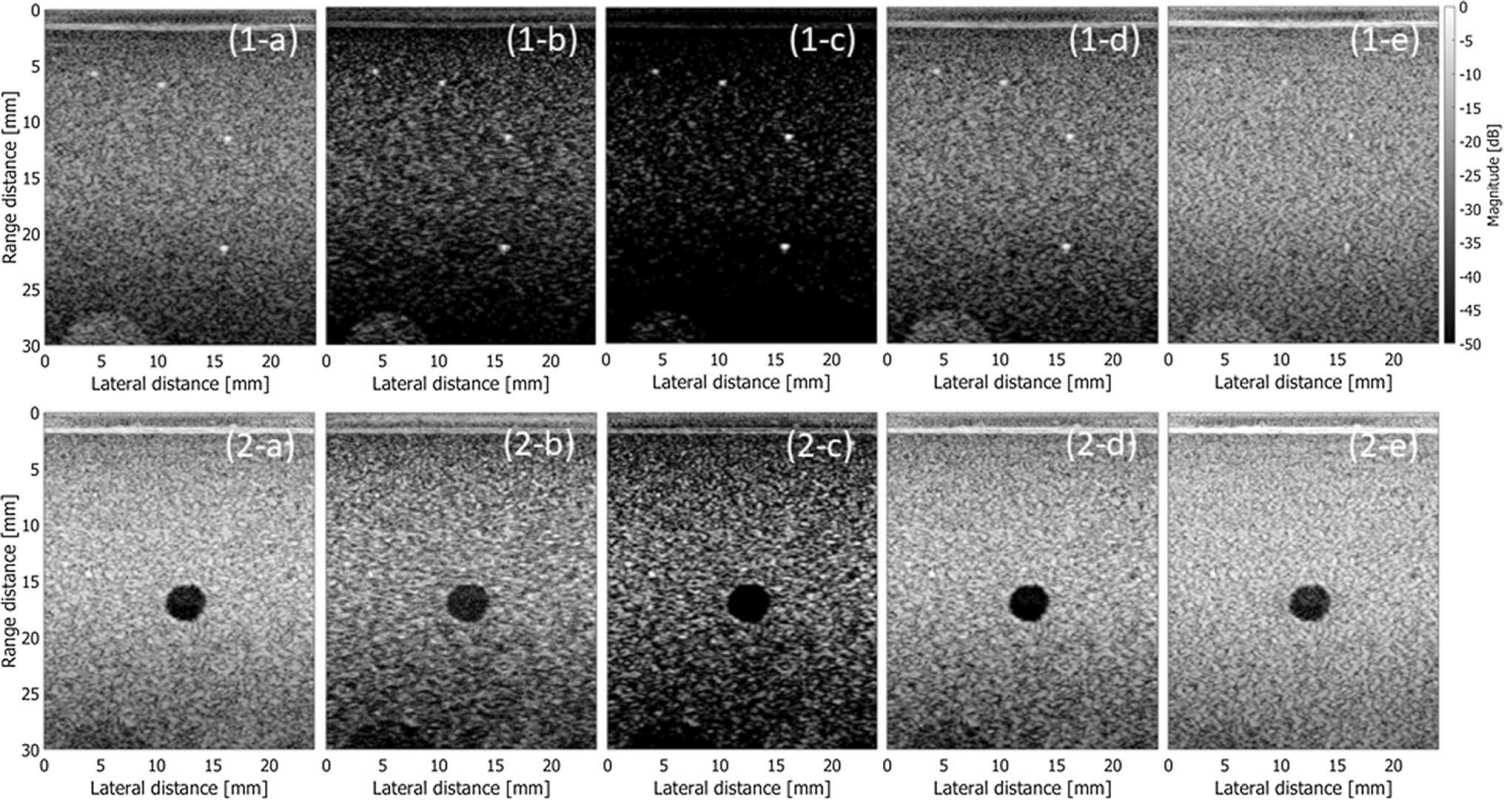
B-mode images of string (1) and cyst (2) phantoms obtained with different beamforming methods. **a** DAS. **b** FDMAS. **c** DAS with CF. **d** DAS with SNR factor (*γ* = 500). **e** MV (sub-aperture size: 2/3 of total aperture, diagonal loading: 0.1 of received power)

Figures 3(1-a) to (1-e) show B-mode images of string targets obtained by DAS, FDMAS, DAS with CF, DAS with SNR factor, and MV, respectively. Figures 3(2-a) to (2-e) show similar results on an anechoic cyst target. Those images were obtained by coherent plane-wave compound imaging with a 7.5-MHz linear array at an element pitch of 0.2 mm [37]. Plane waves were emitted at steering angles from −10 to 10 degrees at angle intervals of 0.5 degrees (21 angles). The *F*-number was set at 2.08, which was calculated from the full width at half maximum (FWHM) of Gaussian apodization. Gaussian apodization was used in DAS, FDMAS, and DAS with CF. Channel data from an aperture with a width of FWHM of Gaussian apodization was processed for linear regression beamforming [15] for estimation of the SNR factor and MV beamforming. In Fig. 3, each beamforming method was applied to RF signals received by individual transducer elements to obtain beamformed RF signals at each transmit steering angle. Such beamformed RF signals at all steering angles were coherently compounded (summed) to obtain the final beamformed RF signals.

**Fig. 4:**
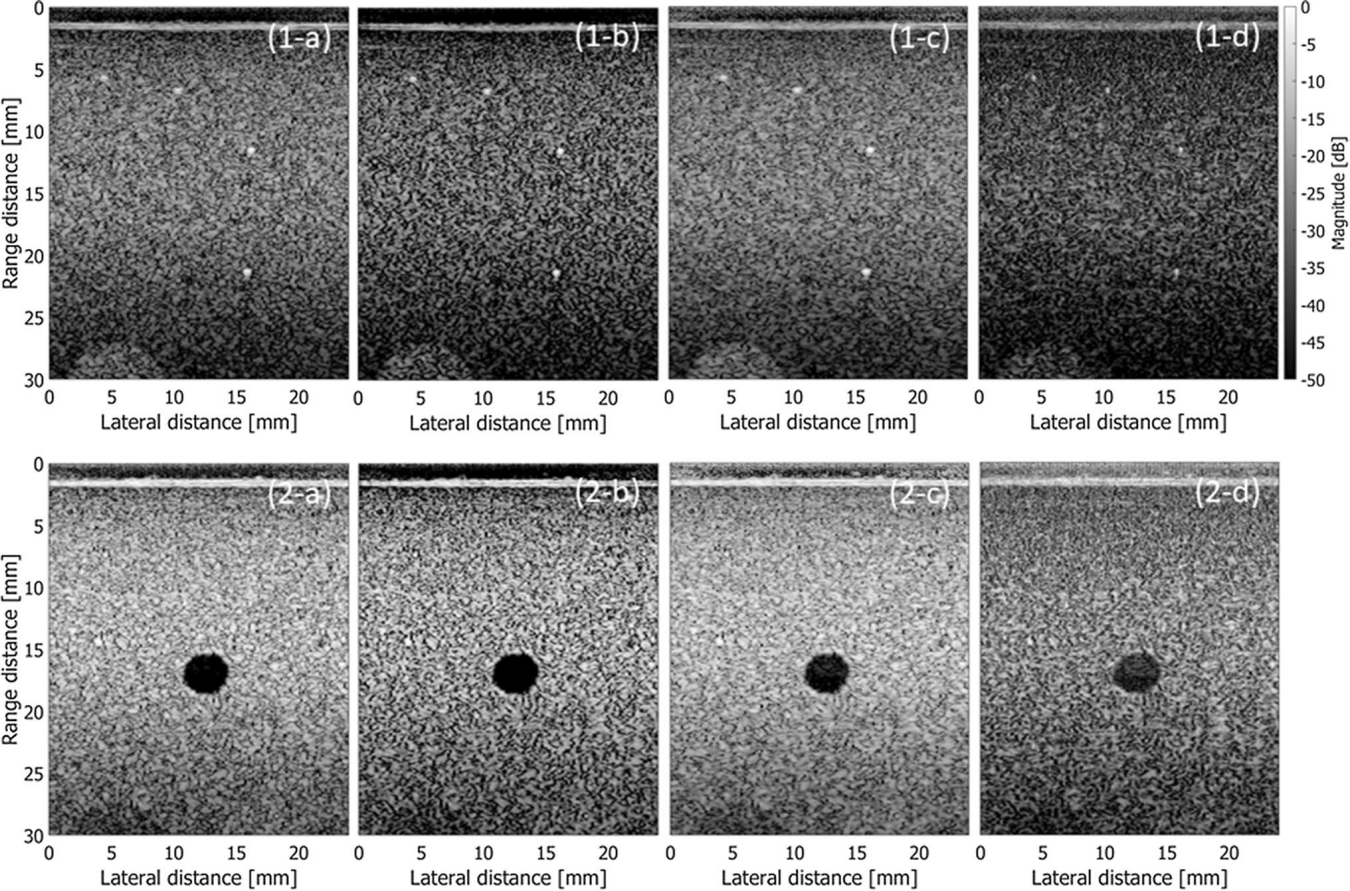
B-mode images of string (1) and cyst (2) phantoms obtained with different methods for coherent compounding. **a** FDMAS. **b** DAS with CF. **c** DAS with SNR factor (*γ* = 500). **d** MV (sub-aperture size: 2/3 of total aperture, diagonal loading: 0.1 of received power)

Figure 4 was obtained by applying FDMAS for compounding beamformed RF signals obtained at different transmit steering angles. Also, DAS with CF, DAS with SNR factor, and MV were used to determine the weights to compound those beamformed RF signals. In Fig. 4, the beamformed RF signal at every transmit steering angle was obtained by DAS beamforming. Then, RF signals obtained at all steering angles were compounded using FDMAS, DAS with CF, DAS with SNR factor, and MV. Figures 4(1-a) to (1-d) show images of string targets obtained with compounding by FDMAS, DAS with CF, DAS with SNR factor, and MV, respectively. Figures 4(2-a) to (2-d) show similar results on a cyst target.

Image quality is in general evaluated using metrics such as spatial resolution, contrast, and CNR [38, 39]. The spatial resolution is often evaluated using the FWHM of an echo from a point (string) target. Contrast and CNR are defined as

15 $${\text{Contrast}} = \frac{{\mu_{\rm {B}} }}{{\mu_{\rm {T}} }},$$

16 $$CNR = \frac{{\left| {\mu_{\rm T} - \mu_{\rm B} } \right|}}{{\sqrt {\sigma_{\rm T}^{2} + \sigma_{\rm B}^{2} } }},$$

where $\mu_{\rm {T}}$ and $\sigma_{\rm {T}}^{2}$ are the mean and standard deviation of amplitudes of beamformed echo signals in a target region, and $\mu_{\rm {B}}$ and $\sigma_{\rm {B}}^{2}$ are those in a background speckle region.

**Fig. 5:**
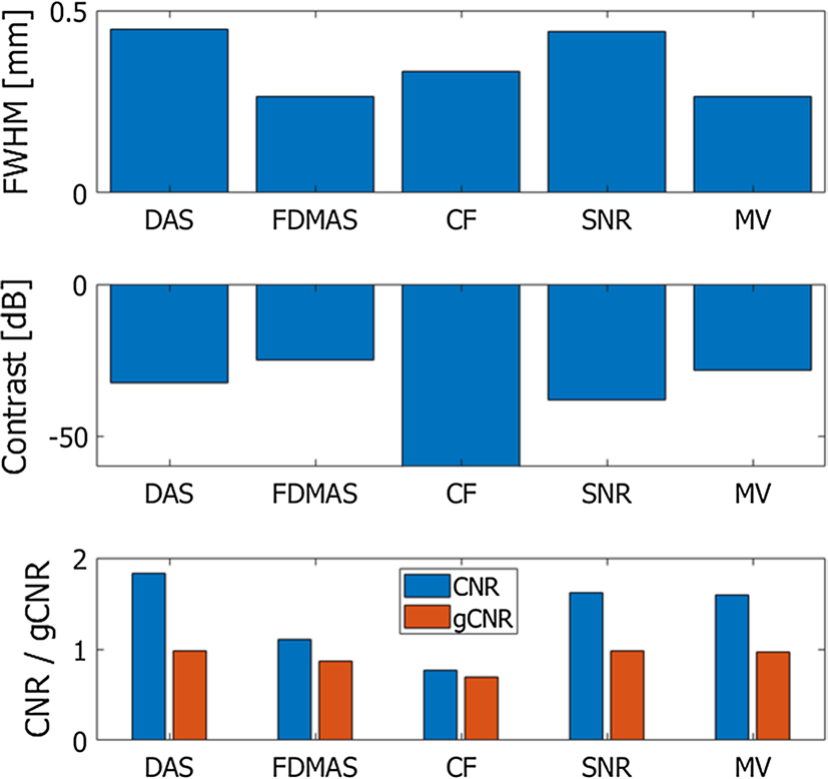
Evaluation metrics obtained from the images in Fig. 3

**Fig. 6:**
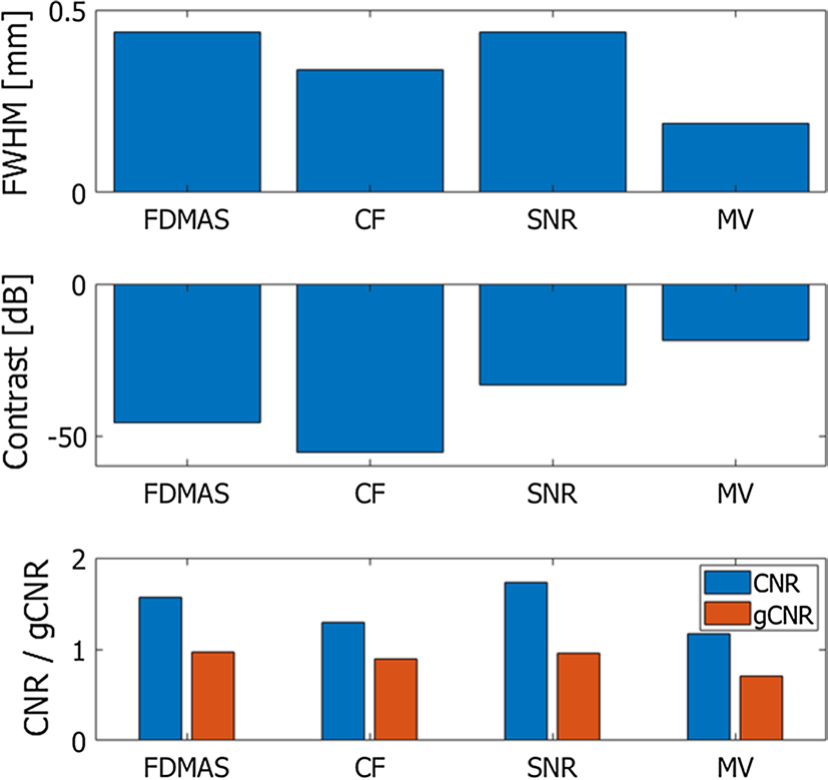
Evaluation metrics obtained from the images in Fig. 4

**Fig. 7:**
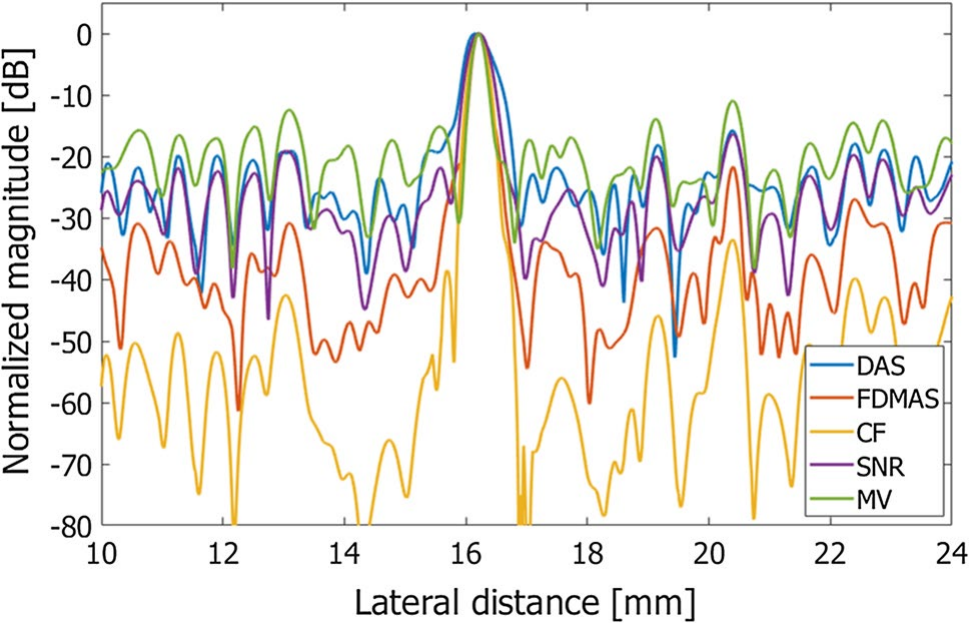
Lateral amplitude profiles of an echo from a string target at a depth of about 10 mm obtained with different beamforming methods

**Fig. 8:**
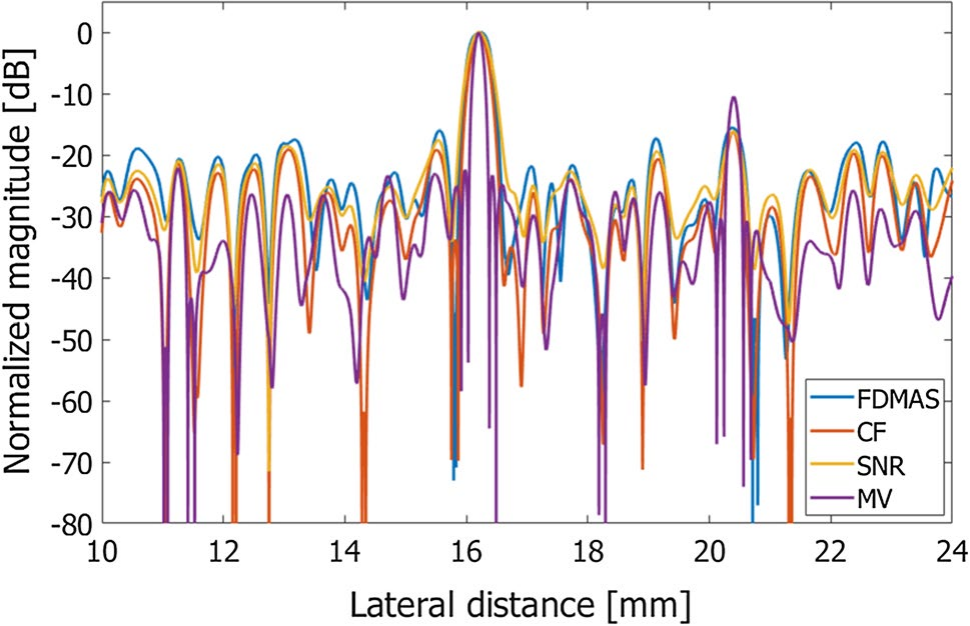
Lateral amplitude profiles obtained with different methods for coherent plane-wave compounding

The metrics described above were evaluated with respect to the images shown in Fig. 3 and summarized in Table 1 and Fig. 5. Also, Table 2 and Fig. 6 summarize metrics evaluated with respect to the images in Fig. 4. Figures 7 and 8 show lateral amplitude profiles of echoes from a string target at a depth of about 10 mm in Figs. 3 and 4, respectively. The lateral resolution is improved by adaptive imaging methods, i.e., the weighting-based methods and MV beamformer. Also, the contrast value was improved by adaptive methods, except for the MV beamformer. On the other hand, CNR was degraded by the adaptive methods.

**Table 1:** Evaluation metrics obtained by different beamforming methods

	FWHM [mm]	Contrast [dB]	CNR	gCNR
DAS	0.449	− 32.37	1.830	0.984
FDMAS	0.264	− 24.84	1.110	0.869
CF	0.332	− 67.62	0.767	0.690
SNR	0.441	− 37.91	1.622	0.986
MV	0.265	− 28.19	1.600	0.966

**Table 2:** Evaluation metrics obtained by different methods for coherent compounding

	FWHM [mm]	Contrast [dB]	CNR	gCNR
FDMAS	0.440	− 45.72	1.571	0.963
CF	0.335	− 55.42	1.297	0.893
SNR	0.438	− 33.16	1.736	0.961
MV	0.189	− 18.50	1.166	0.710

As described in “Coherence-based adaptive imaging”, the weighting-based adaptive imaging methods suppress beamformed echo signals with low coherence. As a result, the contrast between partially coherent (speckle background) and incoherent (anechoic cyst) regions is increased. This could alter the dynamic range of the beamformed signals. As can be seen in Fig. 3(2-c), speckles in B-mode images obtained by the weighting-based method seem “well-resolved.” However, such an effect is caused by the change in signals’ dynamic range. Improvement in the spatial resolution can also be caused by such an effect. As described above, adaptive imaging methods can alter signals’ dynamic range and speckle statistics [40, 41], and such effects damage speckles. This means that a clinically relevant metric, CNR, is degraded significantly.

Several studies have been conducted to deal with such effects of the adaptive imaging methods and evaluate image quality more appropriately. Mallart and Fink [8] and Liu and Waag [42] introduced the van Cittert-Zernike (VCZ) theorem [43] to evaluate the spatial coherence of backscattered ultrasonic waves. The spatial coherence is evaluated by correlation of the channel signal and is sensitive to factors degrading image quality, such as phase aberration, off-axis and reverberation clutters, and thermal noise. Owing to such a characteristic of the spatial coherence, Long et al. proposed a method for evaluation of image quality using lag-one spatial coherence estimated from channel echo signals [44]. The spatial coherence is evaluated as

17 $$R\left( m \right) = \frac{1}{M - m}\mathop \sum \limits_{i = 1}^{M - m} \frac{{\mathop \sum \nolimits_{{n = n_{1} }}^{{n_{2} }} s_{i} \left( n \right)s_{i + m} \left( n \right)}}{{\sqrt {\mathop \sum \nolimits_{{n = n_{1} }}^{{n_{2} }} s_{i}^{2} \left( n \right)\mathop \sum \nolimits_{{n = n_{1} }}^{{n_{2} }} s_{i + m}^{2} \left( n \right)} }}$$

18 $$\widehat{R}\left( m \right) = \left\langle {R\left( m \right)} \right\rangle ,$$

where $n_{1}$ and $n_{2}$ define the axial number of sampled signals used for estimation of the lag-one coherence, and $\left\langle \cdot \right\rangle$ denotes averaging in an assigned region of interest (ROI). The lag-one coherence is obtained by setting $m$ at 1. To obtain the traditional metrics, i.e., contrast and CNR, two regions of interest corresponding to target and speckle background regions are required, and it is preferable that those regions are homogeneous. Therefore, the traditional contrast and CNR are in general evaluated in phantom experiments because it is in general difficult to find a homogeneous region in real tissue. The lag-one coherence can be evaluated in a single ROI as a measure of acoustic clutter and thermal noise.

**Fig. 9:**
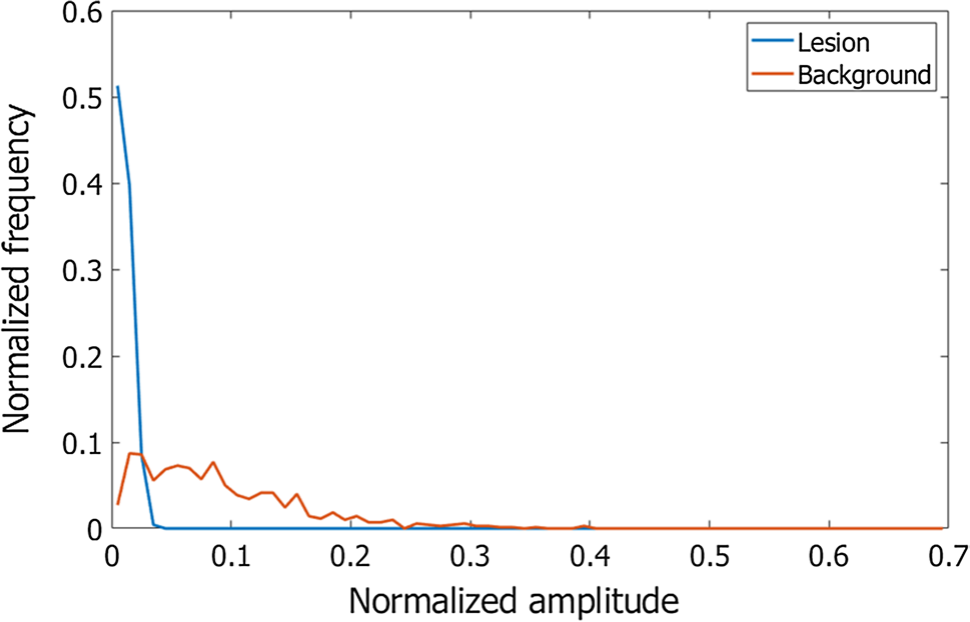
Examples of probability density distributions of echo amplitudes in lesion (cyst) and background regions

Rodriguez-Molares, et al. proposed another metric, namely, generalized CNR (gCNR) [45]. Adaptive imaging methods often improve image contrast significantly, which contributes to lesion detectability. On the other hand, they also damage speckles. Therefore, CNR is regarded as a more clinically important metric for evaluation of image quality. However, just compressing echo amplitude values using sigmoid curve can improve CNR when the traditional definition of CNR given by Eq. (16) is used [45]. The gCNR was developed as a metric that is less influenced by such alteration of signals’ dynamic range. The gCNR is obtained by analyzing the probability density distributions of echo amplitude values in target and background speckle regions and is defined as

19 $$gCNR = 1 - OVL,$$

where $OVL$ is the area of the overlap region between both probability density distributions. Figure 9 shows examples of histograms of echo amplitudes in lesion (cyst) and background regions in the B-mode images shown in Fig. 3. The evaluated gCNRs are summarized in Tables 1 and 2. The gCNR can be used to evaluate the target detectability independently of alteration of the dynamic range [45].

## Recent trends in adaptive imaging

### Coherence-based method

Among the adaptive imaging methods described in “Beamforming”, short-lag spatial coherence (SLSC) imaging is a method that more pays attention to CNR [46, 47]. The lag-one coherence is recently used for evaluation of image quality, but the SLSC imaging methods utilize the spatial coherence described in Eq. (17) at multiple lags. SLSC $R_{\rm {SLSC}}$ is obtained as follows:

20 $$\widehat{R}_{\rm {SLSC}} = \mathop \sum \limits_{m = 1}^{{M_{\rm {SL}} }} R\left( m \right),$$

where $M_{\rm {SL}}$ determines the number of lags used for estimation of SLSC. The SLSC imaging method directly maps SLSC ${R}_{\rm {SLSC}}$ evaluated by Eq. (20), unlike a B-mode image, which maps amplitudes of echo signals. An SLSC image provides a better CNR than a conventional B-mode image, depending on the number of lags used for evaluation of SLSC. A better CNR value can be obtained by SLSC because the variance in the spatial coherence function at short lags is low in a diffuse scattering medium. Recently, it has also been shown that SLSC has potential for use in tissue characterization [48]. On the other hand, SLSC values, which do not show differences in scattering strengths, are directly mapped as described above, and it is still unclear whether SLSC images can replace B-mode images.

### Clutter reduction

Another way to improve image quality without degrading CNR and damaging speckles is to reduce clutter signals. Clutter signals, such as off-axis and reverberation echoes, significantly degrade image contrast. Among the recent developments in ultrasonography, harmonic imaging is the most powerful and widely implemented approach to reduce clutter signals [49–51]. The harmonic imaging approach significantly reduces clutter signals, but clutter signals are still not eliminated perfectly. Byram et al. proposed a method, called aperture domain model image reconstruction (ADMIRE), for reduction of clutter signals contained in channel echo signals [52–54]. In their method, a model of the channel echo signal is expressed as

21 $$g\left( {x;t, \omega } \right) = \mathop \sum \limits_{n = 0}^{N - 1} A\left( {x; x_{n} , z_{n} , \tau_{n} , \omega } \right)e^{{jk\tau \left( {x; x_{n} , z_{n} , \tau_{n} } \right)}} ,$$

where $x$, $t$, and $\omega$ are the lateral position in the aperture, time, and angular frequency, respectively, $k$ is the wavenumber, $x_{n}$ and $z_{n}$ are the lateral and axial positions of a scatterer at time $\tau_{n}$, respectively, $\tau \left( {x; x_{n} , z_{n} , \tau_{n} } \right)$ is the time delay of the wavefront of a signal arriving from a point, which is located at lateral and axial positions of ${x}_{n}$ and ${z}_{n}$, respectively, at time ${\tau }_{n}$, $N$ is the number of scatterers, and $A\left(x; {x}_{n}, {z}_{n}, {\tau }_{n}, \omega \right)$ is the amplitude modulation induced by windowing in Fourier transform and element directivity. The ADMIRE method determines coefficients for fitting the model to the measured signals, and the components estimated from the models for positions outside an acceptance region are discarded as clutter signals. Significant reduction of clutter signals could be achieved by the ADMIRE method in simulation, phantom, and in vivo studies.

Morgan et al. also decomposed received echo signals into components from main lobe, side lobe, and incoherent noise using models of their covariance model, namely, constituent covariance models [55, 56]. The model of the covariance matrix $\widehat{{\varvec{R}}}$ of the received echo signal is expressed as

22 $$\widehat{{\varvec{R}}} = \mathop \sum \limits_{i = 1}^{P} \alpha_{i}^{2} {\varvec{A}}_{i} + {\varvec{N}},$$

where $P$ is the number of constituent components, ${\varvec{A}}_{i}$ is the constituent covariance model of the $i$-th component, $\alpha_{i}^{2}$ is the scalar variance, which corresponds to the power of the $i$-th component, and ***N*** is a noise matrix. The least-square estimate of the scalar variance of each component is obtained as

23 $$\widehat{{\varvec{\alpha}}}_{ls}^{2} = \arg \mathop {\min }\limits_{{{\varvec{\alpha}}^{2} }} {\varvec{R}} - \mathop \sum \limits_{i = 1}^{P} \alpha_{i}^{2} {\varvec{A}}_{iF}^{2} ,$$

where ${\varvec{\alpha}}^{2}$ is the $P \times 1$ vector of variances ${\varvec{\alpha}}^{2} = \left[ {\alpha_{1}^{2} , \alpha_{2}^{2} , \ldots , \alpha_{P}^{2} } \right]^{T}$, and (_F_) and ^T^ denote the Frobenius norm and transpose, respectively. The square root of the estimated variance of the main-lobe component was mapped to obtain a MIST (multi-covariate imaging of sub-resolution targets) image. Significant improvements in contrast and CNR as a result of the MIST method were shown in simulation, phantom, and in vivo studies.

### Estimation of speed of sound

An inhomogeneity in sound speeds in tissue also degrades ultrasonic image quality. Although ultrasonic computed tomography (USCT) was developed for estimation of the spatial distribution of sound speeds [57–62], it is not suitable for ultrasonography based on the pulse-echo method because USCT basically requires measurement of an ultrasonic wave transmitted through a medium. Recently, USCT in the pulse-echo mode [63–66] is being studied intensively.

In the pulse-echo mode, an attempt to estimate the sound speed was made using the difference in propagation time delays of echoes from a target (or target region) obtained with two crossed beams arranged in two directions [67–70] or multiple directions [71, 72]. The arrival time of an ultrasonic echo was estimated from the temporal position of the pulse or correlation analysis applied to echo obtained from two directions. The sound speed was also estimated using the delay profile of an echo from a distinct scatterer [73].

**Fig. 10:**
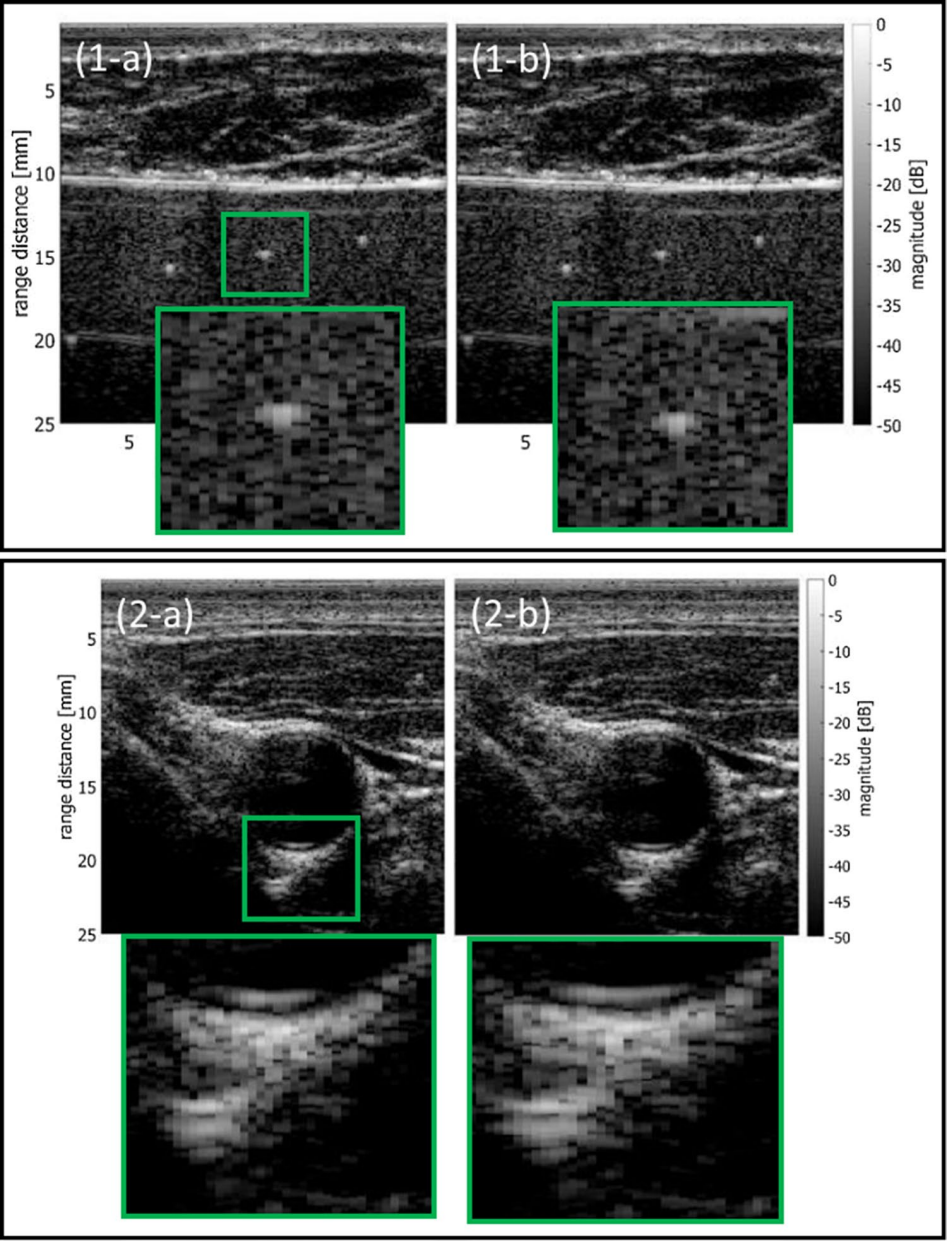
B-mode images of phantom with aberrating layer (1) and carotid artery (2). **a** With constant sound speed of 1540 m/s. **b** With sound speeds estimated by maximizing the coherence factor

In DAS beamforming in pulse-echo mode, it is necessary to assume the sound speed in tissue. Various attempts have been made to estimate the average sound speed to improve the focusing quality in ultrasound beamforming. Ogawa et al. evaluated the magnitudes of the output of a DAS beamformer under different assumed sound speeds to determine the sound speed that maximizes the output [74–76]. A similar method was proposed by Cho et al. [77]. The output of the DAS beamformer becomes maximum when the wavefront of the scattered spherical wave from a receiving focal point is estimated most accurately, i.e., the sound speed is set appropriately. The speed of sound was also determined by evaluating the bandwidth of the beamformed signal in the lateral direction, which corresponds to the lateral resolution of an ultrasonic image [78]. Furthermore, the sound speed was estimated by maximizing spatial coherence evaluated using channel echo signals. The sound speed is estimated by maximizing the CF defined by Eq. (4) [79–83]. An example is shown in Fig. 10. Figures 10(1-a) and (1-b) show B-mode images of a phantom (model 040GSE, CIRS) obtained by DAS beamforming at a constant sound speed of 1540 m/s and with average sound speeds estimated by maximizing the CF [82, 83]. In Fig. 10, the approximate positions of the enlarged regions are indicated by the green rectangles. Echo signals were acquired by the line-by-line sequence with a transmit beam focused at 20 mm. A 7.5-MHz linear array probe was used, and porcine tissue was placed on the top of the phantom as an aberrating medium. The lateral full width at half maximum of the echo from a string target was improved from 0.538 mm to 0.472 mm by correcting the sound speed. Figure 10(2) shows similar results for a human common carotid artery in a transverse plane. As can be seen in Fig. 10(2), echoes from the lumen-intima interface of the posterior wall were visualized in a wider region using the sound speed estimated by maximizing the CF.

### Aberration correction

**Fig. 11:**
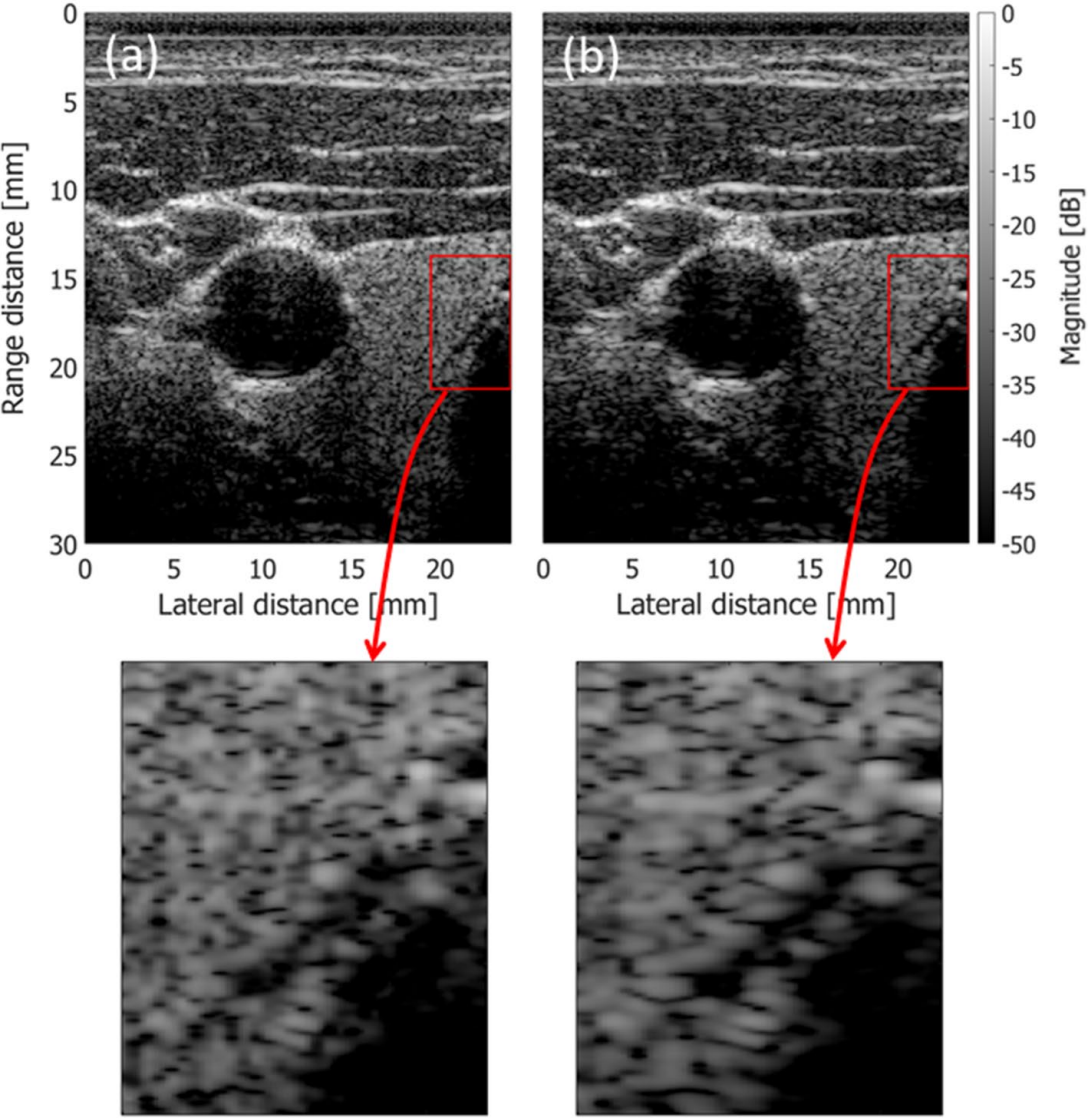
Examples of B-mode images obtained with conventional coherent plane-wave compounding (**a**) and SVD beamforming (**b**)

Inhomogeneity in tissue sound speeds distorts wavefronts of transmitted and reflected ultrasonic waves. Numerous studies have been conducted on measurement and correction of such aberrations [84–88]. Imbault et al. evaluated the spatial coherence based on Eq. (17) and estimated the local sound speed so that the spatial coherence was high in a wide range of lag [89–91]. They used an iterative time reversal focusing method [92] to obtain the aberration profile and create a virtual point reflector to more accurately estimate the spatial coherence in an inhomogeneous medium. Lambert et al. stacked channel echo signals obtained by setting focal points at a point of interest and positions very close to the point of interest [93]. They used the same concept in the iterative time reversal focusing method in estimation of an aberration profile in the reflection matrix method, which also enables suppression of reverberation clutter. In their study, the aberration profile for the point of interest was extracted from those obtained from other focal points using singular value decomposition (SVD) [93]. This concept utilized SVD for aberration correction in coherent plane-wave compounding, called “SVD beamformer” [94]. In SVD beamforming, beamformed complex RF signals in a local area of ${N}_{x}\times {N}_{z}$ samples are obtained from ${N}_{\theta }$ emissions of plane waves steered at different angles. This 3D matrix of a dimension of ${N}_{x}\times {N}_{z}\times {N}_{\theta }$ is reshaped into a 2D Casorati matrix of a dimension of ${N}_{x}{N}_{z}\times {N}_{\theta }$, and then SVD is applied to the 2D matrix. The spatial singular vector with the largest singular value corresponds to the aberration-corrected image. Figures 11a and b show B-mode images obtained with conventional coherent plane-wave compounding and SVD beamforming, respectively. Ultrasonic echoes were acquired with plane-wave emissions at steering angles between -20 and 20 degrees at angular intervals of 0.5 degrees (81 angles). As can be seen in Fig. 9b, the contrast of the thin dark region was improved by correcting aberrations using SVD beamforming.

## Conclusion and perspectives

Since image quality in ultrasonography is a key factor determining the accuracy of diagnosis, numerous studies have been conducted to investigate various phenomena affecting image quality and develop methods for improving image quality, as described in this article. Such investigations on methods for ultrasonic image formation have become increasingly active because programable ultrasound scanners [95–97] became widely available recently. Also, the wide availability of GPUs enables implementations of image formation algorithms that require more intensive computations [98, 99]. Such a research environment should further accelerate explorations in this field, and the deep neural network should also provide a powerful option for medical ultrasound beamforming [100–104], ultrasound image processing such as speckle reduction [105, 106], and image segmentation [107, 108], etc. Through such investigations, ultrasonography will increase its value in medical diagnostics.
